# The Cognitive Deficits Associated with Second-Hand Smoking

**DOI:** 10.3389/fpsyt.2016.00046

**Published:** 2016-03-24

**Authors:** Jonathan Ling, Thomas Heffernan

**Affiliations:** ^1^University of Sunderland, Sunderland, UK; ^2^Northumbria University, Newcastle upon Tyne, UK

**Keywords:** passive smoking, memory impairment, second-hand smoke, everyday memory

Exposure to second-hand smoke (SHS), also known as “passive smoking,” refers to a situation where a non-smoker inhales another person’s smoke either by sidestream or by mainstream exposure to tobacco smoke. Previous research has suggested that not only is prolonged exposure to SHS associated with a range of health-related problems similar to those found in smokers (1, 2) but is also linked to detrimental effects upon cognitive performance in children, adolescents, and adults. For example, children exposed to SHS show reduced vocabulary and reasoning skills when compared with non-exposed children (3) as well as more general cognitive and intellectual deficits (4). More recently, research using serum cotinine as a biomarker of exposure to SHS found that higher levels of serum cotinine were associated with significant reductions in performance in reading, mathematics, and visual and spatial abilities in children and adolescents (5) indicating that higher levels of SHS exposure is associated with poorer cognitive performance. In adults, exposure to SHS in those who had no history of smoking showed significantly reduced performance in processing speed (how quickly one can process information and perform tasks) and executive function (which includes the ability to organize memory, cognitive flexibility, and problem-solving ability) when compared with non-exposed, never smokers (6, 7). In addition, never smokers who lived with smokers for several decades showed a 30% increase in their risk of dementia (8). Recent work has also revealed everyday memory impairments in never smokers with a history of living with smokers for several years; for example, deficits in everyday prospective memory (memory for future actions), such as remembering to carry out everyday activities, keeping appointments with others, or remembering to post a letter on time (7, 9). What is less clear is the mechanism by which SHS might compromise cognitive performance.

The mechanisms underlying the links between SHS exposure and poorer cognitive performance are far from clear. One potential explanation derives from the notion that the carbon monoxide (CO) in tobacco smoke may interfere with the oxygen being delivered to the brain *via* the blood system. CO binds to human hemoglobin more than oxygen does; therefore, it is feasible that by inhaling tobacco smoke with a high level of CO across a prolonged period of time may diminish the amount of oxygen being carried to the brain, which may, in turn, lead to the range of cognitive impairments observed in the previous research. Although this hypothesis is somewhat speculative, it is a viable explanation and future work could test this by measuring levels of CO in the blood of never smokers who have been exposed to SHS and comparing these with never smokers with no history of such exposure. A second possible explanation stems from recent animal research, which has exposed animals to varying degrees of toxic mixtures of chemicals found in tobacco smoke. This research suggests that tobacco-specific procarcinogens may lead to reduced neuronal mass in specific regions of the brain associated with learning and memory; for example, in the hippocampal region of the brain, which is known to be involved in the mediation of memory and learning (10). Future research should therefore measure levels of procarcinogens, such as NKK, in SHS exposed individuals and whether these are associated with increasing cognitive deficits. A third possibility is that prolonged exposure to SHS may lead to a build-up of cardiovascular disease (CVD), which in turn may lead to a range of health and cognitive problems in later life. There is epidemiological evidence that shows prolonged exposure to SHS is associated with a greater increase in CVD (2, 11, 12). A longitudinal design could elucidate this association by observing long-term exposure to SHS and a potential build-up of CVD and how these correlate with performance upon a range of cognitive measures.

Several limitations need to be considered when interpreting research in this area and designing future studies. The exposure to SHS in many studies is based on self-report measures, which may be subject to recall bias and lead to over- or underestimation of exposure. Biological assays could be used to provide more reliable estimates of SHS exposure, for example, cotinine residue levels or nicotine residue in saliva or hair samples. The use of other drugs should also be documented along with the exposure to SHS, because a large body of evidence now exists that documented the health and cognitive risks associated with the use of, for example, excessive amounts of alcohol, ecstasy, cannabis, and other substances. Other factors, such as socioeconomic status and personality variables such as impulsivity and risk-taking propensity, can impact upon health and cognition, so these too should be controlled for in future research.

In summary, the health problems associated with exposure to SHS in individuals who have no history of smoking are fairly well documented. More recently, studies have pointed to a range of cognitive consequences associated with SHS exposure, including everyday memory impairments. Our understanding of what the underlying mechanism(s) are that might account for such ­cognitive deficits is in its infancy, and there is a great deal of scope for research that looks at the relationship between prolonged exposure to SHS, putative physical and neural damage, and the range of cognitive deficits documented in the literature. Given recent concerns raised by health bodies, such as the World Health Organization, about the risk SHS poses to children and adults around the world, it is clear that this is a topic that warrants further research.

## Author Contributions

All authors listed, have made substantial, direct, and intellectual contribution to the work, and approved it for publication.

## Conflict of Interest Statement

The authors declare that the research was conducted in the absence of any commercial or financial relationships that could be construed as a potential conflict of interest.
